# Pretreatment of Glucose–Fructose Syrup with Ceramic Membrane Ultrafiltration Coupled with Activated Carbon

**DOI:** 10.3390/membranes14030057

**Published:** 2024-02-23

**Authors:** Fangxue Hang, Hongmei Xu, Caifeng Xie, Kai Li, Tao Wen, Lidan Meng

**Affiliations:** 1 Light Industry and Food Engineering College, Guangxi University, Nanning 530004, China; hangfx@163.com (F.H.); 13198088386@163.com (H.X.); fcx11@163.com (C.X.); gxlikai@gxu.edu.cn (K.L.); 2 Guangxi Zhuang Autonomous Region Product Quality Inspection Research Institute, Nanning 530004, China; wantao530000@126.com; 3 Light Industry and Chemical Engineering College, Guangxi Vocational & Technical Institute of Industry, Nanning 530004, China

**Keywords:** ceramic membrane, ultrafiltration, glucose–fructose syrup, membrane fouling, cleaning

## Abstract

Ceramic membranes are applied to remove non-sugar impurities, including proteins, colloids and starch, from glucose–fructose syrup that is dissolved from raw sugar using acid. The performance of ceramic membranes with 0.05 μm pores in clarifying high-fructose syrup was investigated under various operating conditions. The flux decreased rapidly at the start of the experiment and then tended to stabilize at a temperature of 90 °C, a transmembrane pressure of 2.5 bar, and cross-flow velocity of 5 m/s under total reflux operation. Moreover, the steady-state flux was measured at 181.65 Lm^−2^ h^−1^, and the turbidity of glucose–fructose syrup was reduced from 92.15 NTU to 0.70 NTU. Although membrane fouling is inevitable, it can be effectively controlled by developing a practical approach to regenerating membranes. Mathematical model predictions, scanning electron microscopy, energy dispersive X-ray spectroscopy, and Fourier-transform infrared spectroscopy revealed that foulants primarily responsible for fouling are composed of polysaccharides, proteins, sucrose, phenols, and some metal elements, such as calcium, aluminum, and potassium. Due to the removal of suspended colloidal solids, the membrane-filtered glucose–fructose syrup was decolorized using activated carbon; the filtration rate was effectively improved. A linear relationship between volume increase in syrup and time was observed. A decolorization rate of 90% can be obtained by adding 0.6 (*w*/*w*) % of activated carbon. The pretreatment of glucose–fructose syrup using a ceramic membrane coupled with activated carbon results in low turbidity and color value. This information is essential for advancing glucose–fructose syrup and crystalline fructose production technology.

## 1. Introduction

Membrane separation technology (MST) uses membrane selective permeation to achieve the separation, purification, and concentration of different components of a liquid material. MST has been widely used in many fields, such as wastewater treatment, juice clarification, and pharmaceuticals, due to its versatility, low energy consumption, and high efficiency. Membrane filtration can provide high-quality juice with reduced turbidity, low viscosity, and significant color removal. The application of MST in the sugar industry has been studied since the early 1970s [1,2,3]. The research and application of MST in the sugar industry has expanded in recent years. Apart from clarifying mixed juice, MST is also applied to other materials, including limed juice, raw sugar remelt syrup, brown sugar remelt syrup, and molasses [4,5,6,7,8,9,10,11,12,13]. Polyethersulphone (PES) membranes with a molecular weight cutoff of 15–50 kDa and mineral membranes for treating 50 °Bx remelt syrup has shown significant results. The color value can be reduced by 50%, with increased purity, effectively removing non-sugar impurities, such as suspended solids, pigments, and colloids, from the return syrup. However, when using metal membranes with a molecular weight cutoff of 1 kDa to treat 48 °Bx remelt syrup, a steady-state flux of 29 Lm^−2^ h^−1^ and a 58.67% pigment removal rate were achieved. The membrane’s decolorization effect is moderate, requiring combining with ion exchange resin to achieve a higher decolorization rate. Despite good impurity removal and decolorization effects, organic and metal membranes exhibit relatively low flux [10,14]. The studies have yielded positive results, indicating a promising future for MST in the sugar industry.

Glucose–fructose syrup and crystalline fructose have a unique flavor and are thus widely used in food and beverage as sweeteners [15]. The production of glucose–fructose syrup and crystalline fructose using raw sugar as the primary ingredient offers the potential to diversify sucrose products and enhance the market competitiveness of sucrose. Due to the high levels of protein, colloids, starch, and other impurities present in raw sugar, the manufacturing of syrup viscosity becomes challenging, exhibiting low filtration efficiency and increased consumption of activated carbon. Therefore, before the activated carbon decolorization process, an impurity removal process should be added to remove colloidal impurities in raw sugar. Ceramic membranes, precisely engineered filters sintered from Al_2_O_3_, TiO_2_, or ZrO_2_ at ultra-high temperatures, are widely used to physically remove particles ranging from 0.005 to 10 μm in liquids. Their high physical strength and chemical and thermal stability all contribute to their effectiveness [16]. Furthermore, ceramic membranes are recognized as advanced physical separation technology, offering high separation efficiencies, high temperature and pressure tolerance, and antimicrobial properties. It is also widely used in diverse industries, including the environment, food, and chemical sectors.

Membrane separation performance is not as efficient as it has always been, and due to the presence of colloidal impurities, membranes are subject to varying degrees of contamination during filtration. This condition is frequently observed on the external surfaces, at pore openings, or within pores of membranes. Ceramic ultrafiltration membranes with a pore size of 0.2 μm are used for filtering clarified juice (lime defecation) in sugar mills with good clarification (low turbidity, low viscosity), but changes in permeate flux are not mentioned because dead-end filtration leads to severe membrane contamination [17]. The treatment of liming–sulphitation juices with polymeric spiral wound membranes was also described. However, the membranes were easily contaminated, and the average flux dropped sharply to 7 Lm^−2^ h^−1^ [12]. Therefore, it is necessary to study the cleaning and regeneration methods of membranes.

Ceramic membranes typically possess corrosion resistance, high-temperature stability, efficient filtration, a long lifespan, and easy cleaning features. In the early stages of research, we found that ceramic membranes are suitable for filtering different raw sugarcane juice materials, demonstrating highly stable flux, impurities removal and decolorization effects [18,19]. So, in this study, ceramic membrane separation technology was used to pretreat the glucose–fructose syrup hydrolyzed from raw sugar to remove colloidal impurities and reduce the problem of pore size blockage and a large consumption of activated carbon. We aim (i) to evaluate the flux and effects of clarification on ceramic membranes, (ii) to establish a model for the investigation of the membrane fouling mechanism, (iii) to investigate the membrane fouling mechanism, and (iv) to identify an effective membrane cleaning method.

## 2. Materials and Methods

### 2.1. Experimental Materials

The ceramic membrane used was provided by Jiangsu Jiuwu Hi-Tech Co., Ltd., Nanjing, Jiangsu, China. The specifications of the membrane are provided in Table 1. Raw sugar was collected from a local sugar mill (Guangxi, China). The production steps of glucose–fructose syrup were as follows: raw sugar → dissolution → acidolysis → pH value adjusting →glucose–fructose syrup dilution (used as the feed for the experiment) → ceramic membrane filtration. Before entering the ceramic membrane filtration system, the glucose–fructose syrup was filtered using a 200-mesh screen to remove residual insoluble particles. It was heated to 90 °C in a steamer as feed for the experiment.

### 2.2. Experimental Setup

**Figure 1:**
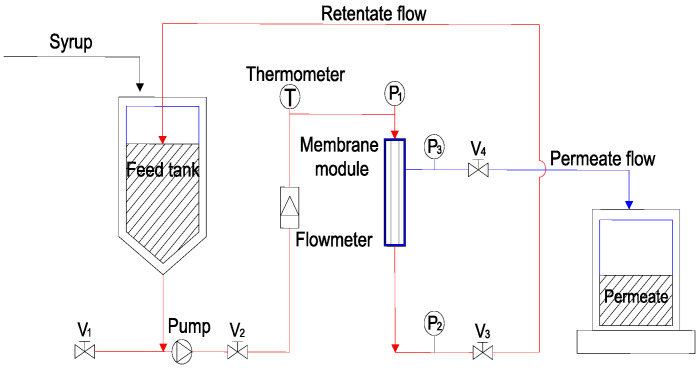
Schematic of the experimental setup.

Figure 1 depicts the schematic of the experimental setup utilized in this study. The glucose–fructose syrup was introduced into a feed tank and subsequently pumped into the membrane module for radial permeation through the membrane under pressure, resulting in clarification. The retentate was recirculated to the feed tank, while the permeate was collected in separate containers. Permeate flux changes over time were measured using a stopwatch and cylinder.

### 2.3. Experimental Methods

The operating conditions employed for ultrafiltration membrane clarification are presented in Table 2. Feed circulation between the feed tank and membrane module was facilitated by the feed pump, while the flow meter displayed the feed rate. The cross-flow velocity (CFV) is determined as the ratio of the flow meter reading to the filtration area.

Membrane cleaning: After each experiment, the membrane was subjected to in situ cleaning procedures.

After each cleaning operation, the membrane’s water flux is quantified, and the recovery rate is determined. The efficacy of the cleaning procedure was assessed based on the calculated flux recovery rate, employing the following formula:

(1) $$r=\frac{j_{2}}{j_{1}}\times 100\%$$

where:

- r is the flux recovery rate (%);
- j_1_ is the deionized water flux of the new membrane (Lm^−2^ h^−1^); and
- j_2_ is the deionized water flux after cleaning (Lm^−2^ h^−1^).

### 2.4. Analytical Methods

The feed and permeate samples were analyzed for Brix, color, turbidity, and pH [18,19,20]. The analytical methods employed in the assessment of the feed and permeate juice adhered to the protocols recommended by the International Commission for Uniform Methods of Sugar Analysis (ICUMSA).

Brix represents a refractometric measurement of dry matter content. Subsequently, the syrup was filtered using Whatman filter paper, followed by Brix determination utilizing a digital refractometer (PAL-3, Atago, Guangzhou, China).

The syrup color was quantified using a spectrophotometer (722 N, Jingke, Shanghai, China). Each sample, consisting of 30.0 g of solid, was dissolved and made up to a final volume of 100 mL. The resulting sample solution was then transferred into a 1 cm colorimetric cuvette, with the blank point adjusted using deionized water. Absorbance measurements were performed at wavelengths of 420 nm and 720 nm, employing the following equation:

$$X=A_{420}-A_{720}$$

where A_420_ is the absorbance at 420 nm, and A_720_ is the absorbance at 720 nm.

The turbidity of the syrup solutions was quantified using a state-of-the-art digital turbidity meter (HACH, Loveland, CO, USA).

The pH levels of the syrup solutions were determined utilizing a cutting-edge digital pH meter (PHS-3C, Leici, Shanghai, China).

The conductivity of the syrup solutions was measured using a digital conductivity meter (DDS-11A, Haibo, Shenzhen, China), while UV-visible spectrophotometry determined light transmittance. The sample was diluted to a solid content of 30% with distilled water, and absorbance at 420 nm was measured after adjusting the zero point using distilled water as a blank.

The colloid content was determined using the method described by Meng et al. [21]. Scanning electron microscopy (SEM, S-34000N, HITACHI, Tokyo, Japan) was employed to observe the surface and cross-sectional morphology of the membrane filtration layer, utilizing a Phenom Pro instrument from Phenom in Holland. Energy dispersive X-ray spectroscopy (EDX, PV8200, Philips, Amsterdam, The Netherlands) was utilized for elemental composition analysis of the membrane samples. Fourier transform infrared spectroscopy spectrometer (FTIR) (Nicolet 50, Thermo Fisher Scientific, Waltham, MA, USA), was used to analyze impurity functional groups on the contaminated membrane surface.

## 3. Results and Discussions

### 3.1. Influence of Operating Parameters on Flux and Clarification of Glucose–Fructose Syrup

#### 3.1.1. Temperature

**Figure 2:**
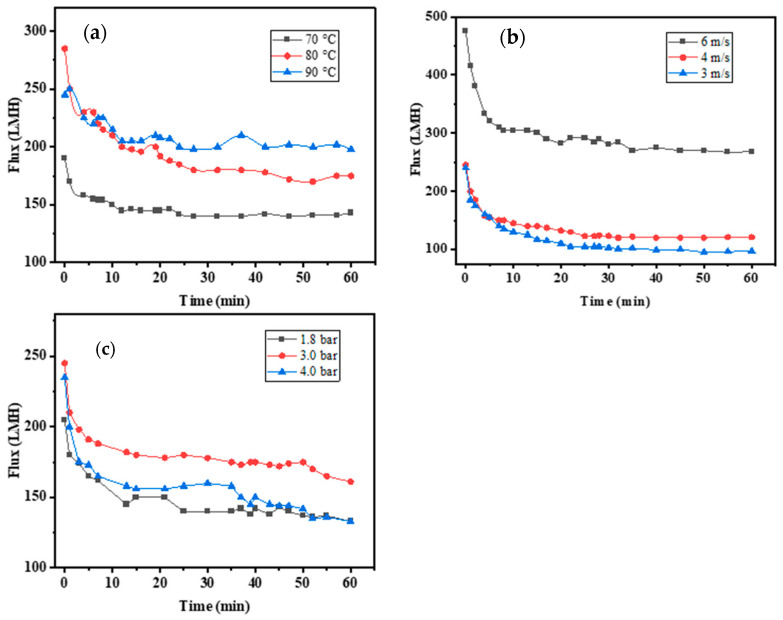
Influence of operating parameters on flux. (**a**) Temporal variation of flux in syrup ultrafiltration. (**b**) Cross-flow velocity variation of flux in syrup ultrafiltration. (**c**) TMP variation of flux in syrup ultrafiltration.

Flux is a significant parameter that measures membrane separation. The temporal variation of the flux during the ceramic membrane ultrafiltration of syrup at a transmembrane pressure (TMP) of 2.5 bar; cross-flow velocity of 5.0 m/s; and temperatures of 70 °C, 80 °C, and 90 °C is shown in Figure 2a. The increase in temperature from 70 °C to 90 °C caused an increase in the steady-state flux from 151.8 Lm^−2^ h^−1^ to 213.2 Lm^−2^ h^−1^ (60 min). The increasing temperature resulted in the syrup’s low viscosity and high mass transfer coefficient. Thus, the quantity of foulants deposited on the membrane surfaces or trapped under the pores was reduced, resulting in a relatively small overall filtration resistance with high flux [22].

The turbidity of the glucose–fructose syrup was reduced from 92.15 NTU to 0.70 NTU, and 99% of the suspended solid particles were removed (Table 3). However, the ceramic membrane with a 0.05 μm pore size only removed about 10% of the pigment. However, the pigment removal effect of the ceramic membrane with a 50 nm pore size was only about 10%, and the electrical conductivity was also similar, indicating that the effects of decolorization and desalting of the ceramic membrane were insignificant. No significant difference was observed between the three groups of permeate. The chromaticity of high-quality permeate syrup at 90 °C is slightly higher than the low temperature, probably due to the longer heating time of the pretreatment process and the fluctuation of temperature instability. The temperature of raw sugar hydrolysis was 90 °C, resulting in a more significant membrane flux. After comprehensive consideration, a final temperature of 90 °C should be selected as the material temperature of membrane filtration.

#### 3.1.2. Cross-Flow Velocity

The temporal variation of the flux during the ceramic membrane filtration of syrup at a TMP of 2.5 bar; a temperature of 90 °C; and cross-flow velocities of 3.0, 4.0, 5.0, and 6.0 m/s is shown in Figure 2b. When the cross-flow velocity was 3 m/s, the initial flux was 240 Lm^−2^ h^−1^, and the steady-state flux was 97 Lm^−2^ h^−1^. When the cross-flow velocity was 4 m/s, the initial flux was 245 Lm^−2^ h^−1^, and the steady-state flux was 121 Lm^−2^ h^−1^. When the cross-flow velocity was 6 m/s, the initial flux was 475 Lm^−2^ h^−1^, and the steady-state flux was 268 Lm^−2^ h^−1^. The steady-state flux increased at cross-flow velocities of 3–6 m/s. The filtration velocity was large, and the shear velocity along the membrane’s surface increased, reducing the thickness of the filter cake layer and concentration polarization because removing particle impurities on the surface of the deposited film decreased the fouling of the membrane.

Table 4 shows the quality indexes of permeate at different cross-flow velocities. Various indexes indicated that using the same membrane pore size to filter the syrup only changed the cross-flow velocity. Except for the change in flux, no significant difference was observed in each index. The increase is dependent on the properties of the material and energy consumption. The maximum flux was obtained at a cross-flow velocity of 6 m/s. At this flux value, power consumption increased, and the demand for the equipment increased. In addition, the flux was 200 Lm^−2^ h^−1^ when the film surface velocity was 5 m/s, which met the requirements. Thus, we selected the operating parameter’s cross-flow velocity of 5 m/s.

#### 3.1.3. Transmembrane Pressure

The temporal variation of the flux during the ceramic membrane ultrafiltration of syrup at cross-flow velocities of 5.0 m/s, a temperature of 90 °C, and TMP values of 1.8, 2.5, 3.0, and 4.0 bar is shown in Figure 2c. The steady-state flux of membrane filtration did not increase with increasing TMP, and the steady-state flux increased when the TMP increased from 0.18 MPa to 0.25 MPa. However, the steady-state flux decreased when the TMP increased to 0.30 MPa. In the filtration process, the cake layer was pressed under pressure, and the filtration resistance increased; thus, the membrane flux decreased. Table 5 shows the indexes of permeate under different TMPs. No significant difference was observed in each index. The optimal TMP was 0.25 MPa.

#### 3.1.4. Flux Stability of Ceramic Membrane

**Figure 3:**
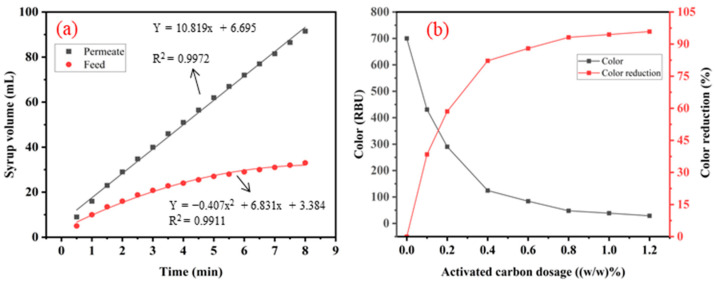
(**a**) Comparison of feed and permeate filtration rates. (**b**) Decolorization of membrane-filtered glucose–fructose syrup using activated carbon.

Figure 3a shows that the temporal variation in volume increases with 200 mL feed and permeate were filtered with 0.1% active carbon and an equal amount of perlite under normal pressure. A linear relationship between the volume increase in the syrup and time was observed. When filtration reached 8 min, the permeate collected was 86.6 mL of clear liquid. However, the filtration process of the feed slowly increased, and only 24.5 mL was filtrated at the same time. Then, an increase of 1 mL per 30 s was achieved when the steady state was reached. Therefore, after filtration through the ceramic membrane, the glucose–fructose syrup was decolorized through activated carbon, and the filtration rate was improved. Figure 3b shows the trend of decreasing color value with increasing activated carbon addition. As can be seen from the figure, when the additional amount of activated carbon reaches 0.6 (*w*/*w*) %, the removal rate of color is close to 90%, and the change efficiency is relatively low when the additional amount of activated carbon powder is increased. When the addition of activated carbon reaches 1.2 (*w*/*w*) %, the removal rate of syrup color reaches 96%.

When colloidal suspended particles, such as proteins, starch, and polysaccharides, are present in a solution, they can impact activated carbon adsorption. First, colloidal suspended particles may compete with adsorption sites on activated carbon, occupying some adsorption sites and reducing the adsorption efficiency for the target substance. Second, there is a decrease in adsorption capacity; the presence of colloidal particles may reduce activated carbon’s effective adsorption surface area, thereby decreasing the overall adsorption capacity. Third, colloidal particles may adhere to the pore surfaces of activated carbon, causing pore blockage and affecting the ability of solute molecules to enter the pores, thus diminishing the adsorption effectiveness. Finally, the presence of colloidal suspended particles may alter the kinetic characteristics of the adsorption process, such as adsorption rate and equilibrium time. Different colloidal particles may exhibit different affinities, causing activated carbon to adsorb certain particles and preferentially reduce adsorption for other components.

**Figure 4:**
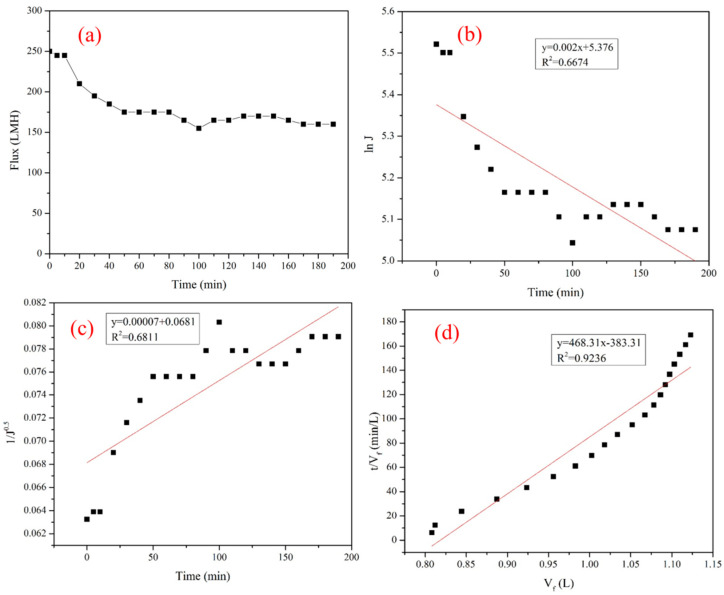
(**a**) Time variation of flux in syrup ultrafiltration. (**b**) Linear fit of the experimental data using the complete pore-blocking model. (**c**) Linear fit of the experimental data using the pore narrowing model. (**d**) Linear fit of the experimental data using the cake filtration model.

The time variation of flux in syrup ultrafiltration is shown in Figure 4a. The ceramic membrane can obtain relatively large membrane fluxes when filtering syrup. The flux declined from 250.0 Lm^−2^ h^−1^ to 160.0 Lm^−2^ h^−1^, and the average flux was 182.6 Lm^−2^ h^−1^ during the experiment conducted for 190 min. Therefore, a ceramic membrane has a high and stable flux, making it suitable for industrial applications.

### 3.2. Fouling Mechanism Models

The comparative results between the flux of the membrane treated with glucose–fructose syrup and the deionized water flux of a new membrane show that the former was lower because the syrup severely fouled the membranes. Thus, it is essential to investigate the mechanism of membrane fouling. Figure 4 shows the fitting relationship among the experimental data of cross-flow velocities at 5 m/s and the experimental data of different mathematical models under the total reflux operation at 90 °C and 0.25 MPa during membrane ultrafiltration of the glucose–fructose syrup. The fouling models were matched with the experimental data of the membrane during glucose–fructose syrup ultrafiltration. The following models were included: complete pore blocking model, pore narrowing model, and cake filtration model. The equations for each of these models are shown in Table 6. Further details on these models can be found in previous studies [18,23,24].

Figure 4b–d illustrate the fitting of the different fouling models corresponding to the flux data for a filtration period of 190 min. The respective linear correlation coefficients (R^2^) of the complete pore blocking, pore narrowing, and cake filtration were 0.6674, 0.6811, and 0.9236, respectively. The linear correlation coefficients revealed that cake filtration prevailed and adequately represented the fouling mechanism during glucose–fructose syrup ultrafiltration.

As a result, the amount of contaminants deposited on the membrane surface or trapped under the pores is reduced. The formation of the cake layer and the degree of contamination caused by pore clogging and pore narrowing are all mitigated, increasing permeate flux. The surface of the cake layer is in direct contact with the host syrup, enhancing the transfer. Therefore, the filter cake layer contamination dominated the three types of contamination [22].

### 3.3. SEM Analysis

**Figure 5:**
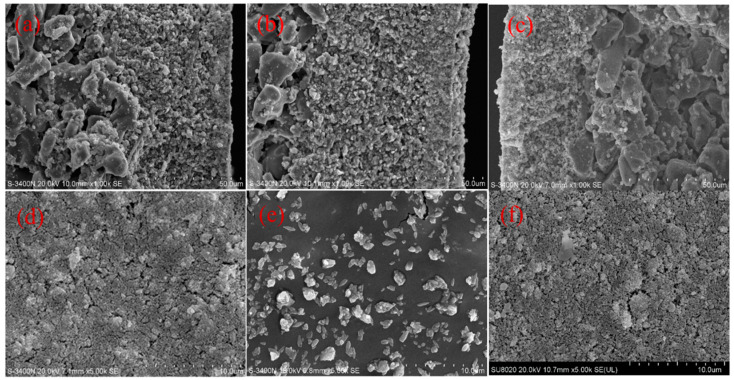
SEM micrographs of the cross-section of the membrane ((**a**): new membrane; (**b**): fouled membrane; (**c**): cleaned membrane) and SEM micrographs of the surface of the membrane ((**d**): new membrane; (**e**): fouled membrane; (**f**): cleaned membrane).

We performed SEM to observe the contaminated status and distribution after the experiment. The contaminated ceramic membranes were filtered at a TMP of 2.5 bar, a CFV of 5.0 m/s, and a temperature of 90 °C for 1 h. The contaminated ceramic membranes were used for SEM measurements to investigate the membrane fouling mechanism. Figure 5a–f show the cross-sectional and surface SEM images of the new, fouled, and cleaned membranes, separately.

Figure 5a shows that the cross-sectional view of the new membranes reveals three different morphological characteristics: the right is the surface coating on the film body and is mainly a separation layer; the middle is the transition layer of the film, and the pore size and thickness are greater than the film layer; and the left is the support layer, where the film material particles are larger, and the roughness is relatively large. It can also be seen from Figure 5b that a 2 μm thick filter cake layer was deposited on the membrane surface. No significant presence of contaminants was observed in the cross-section of the fouled membrane’s separation, transition, and support layers, indicating that the membrane material was less affected. After cleaning, the filter cake on the separation layer of the ceramic membrane was removed, as shown in Figure 5c. After cleaning, the filter cake layer above the membrane layer is no longer present, and the membrane pores are clear.

Figure 5d shows that the surface of the new ceramic membrane appears smooth, and the membrane pores are visible. In comparison, the surface of the contaminated membrane (Figure 5e) is denser, with visible inorganic salt particles on the surface area. The membrane pores are invisible when magnified at the same scale as the new membrane. As shown in Figure 5f, the cleaned ceramic membrane has a surface morphology similar to the new membrane, with clear membrane pores. Compared to the contaminated membrane, the filter cake layer has been removed.

### 3.4. EDX Analysis

**Figure 6:**
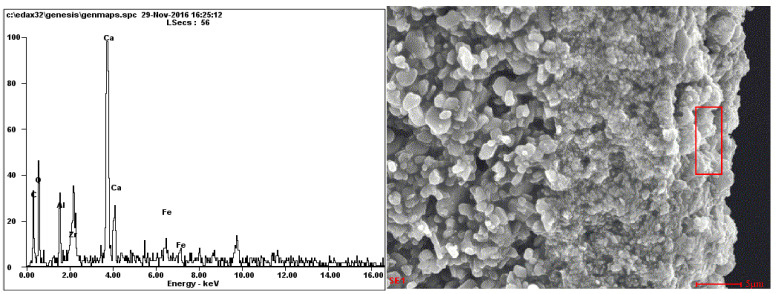
EDX analysis of the cross-sections of fouled membrane layers.

The results of the EDX analysis of the cake layer of the contaminated membrane are shown in Figure 6, while Table 7 corresponds to the analysis of the elemental composition of the contaminants at different locations of the contaminated and cleaned membranes. The EDX analysis results of the fouled membrane indicate that the contamination layer has Al, Zr, O, Ca, Fe, and C. The high content of Ca^2+^ in the material was due to the high hardness of tap water used in the cleaning process. The membrane, which is mainly composed of ZrO_2_, is easily networked by Ca^2+^, forming a CaCO_3_ precipitation that attaches to the surface of the membrane and blocks the membrane holes. Therefore, in the application and cleaning of the ceramic membrane, it is necessary to use 0.5% nitric acid in addition to NaOH solution to ensure that the membrane layer is not affected by Ca^2+^. The proportion of the C elements in the membrane layer of the fouled membrane was significant, indicating that organic matter was present and that the membrane pores were polluted [18]. The transition and support layers have no additional elements except the material of the membrane itself. Contaminants are also present in the transition and support layers in relatively small amounts that do not significantly interfere with the membrane’s filtration performance. In the EDX analysis of the cleaned membrane, no Fe, Ca, Al, or other elements were observed. Zr and some C elements were derived from the materials used to make the ceramic membranes. The content of C elements was reduced, indicating that the cleaning effect of the membrane was improved [18].

### 3.5. FTIR Analysis

**Figure 7:**
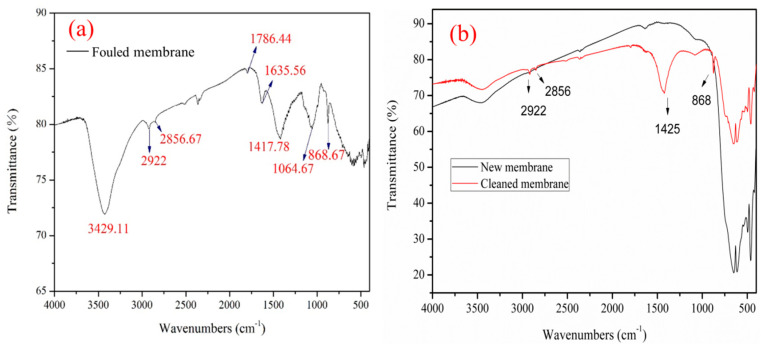
(**a**) FTIR spectra of foulants scraped from the fouled membrane. (**b**) FTIR spectra of scraped from the new membrane and cleaned membrane.

In general, FTIR spectroscopy can provide detailed information about the deposition of biopolymers on the membrane surface. Figure 7 shows the FTIR analysis results of the ceramic membrane surface after it was fouled with syrup. The spectrum showed a broad absorption peak at 3429.11 cm^−1^, indicating the stretching and vibration of the O-H bond in the hydroxyl function groups [18,25]. The wave numbers at 2922 and 2856.67 cm^−1^ were assigned to the symmetry flex vibration of C-H bonds in -CH_3_ and -CH_2_, respectively. These bonds originated from the catenated carbon-containing materials in the glucose–fructose syrup. The wave numbers suggested that organic components had been deposited on the membrane. The wave numbers at 1786.44 cm^−1^ were assigned to carboxylic groups [18,26]. The band at 1417.78 cm^−1^ (methyl stretching) was ascribed to polysaccharides and lipids [18,27]. The band at 1635.56 cm^−1^ (C–N–H stretching) was attributed to the amino group, indicating the presence of proteins [18,19,28,29]. The peak at 1417.78 cm^−1^ represents the C-O stretching of phenols. The bands at 1064 and 868.67 cm^−1^ are attributed to the C–O stretching of alcoholic compounds. The alcoholic C–O bonds may have originated from polysaccharide-like substances [18,25]. The sucrose C-O bonds may have been derived from polysaccharide-like substances in syrup [30]. The peaks displayed from 700 to 400 cm^−1^ in the spectra are attributed to the membrane material [31]. From the FTIR spectra of the new membrane and the membrane after cleaning, it can be observed that vibrations are still present around 2922, 2856.67, and 1417 cm^−1^ after cleaning. This indicates the continued presence of a small amount of organic compounds on the surface of the cleaned membrane. However, vibrations at 1786 and 1635 cm^−1^ have disappeared, indicating that the cleaning of the membrane has been effective. The findings suggest membrane fouling may be attributed to polysaccharides, proteins, aliphatics, phenols, and sucrose.

### 3.6. Membrane Cleaning

The contamination of the filter cake layer is the dominant cause of membrane contamination, as reported in the previous research. Referring to the last experience research, NaOH, NaClO, and HNO_3_ were selected as the regeneration chemicals for the contaminated membrane. As strong bases and oxidizers, NaOH and NaClO have good removal ability for organic pollutants (various macromolecular colloids, suspended impurities, etc.), and sodium hydroxide can loosen, emulsify, and disperse the sediment on the membrane surface to achieve the best cleaning effect. HNO_3_ is a strong inorganic acid that can remove the sediments’ stubborn inorganic salts (calcium and magnesium precipitates) [18,32]. Sodium hypochlorite can achieve the best cleaning effect by stimulating the gel layer on the membrane surface for oxidative decomposition and shedding [33]. The cleaning conditions were determined through repeated experiments. First, we choose to use NaOH and NaClO to remove the organic matter covered on the surface of the pollutant and then use HNO_3_ to remove the metal salts deposited in different parts of the membrane to improve the cleaning effect [18,19]. The contaminated membranes were cleaned sequentially with a mixture of (i) deionized water, (ii) 1% NaOH, (iii) 1% NaOH + 0.5% NaClO, and (iv) 1% NaOH + 0.5% NaClO + 0.5% HNO_3_.

**Figure 8:**
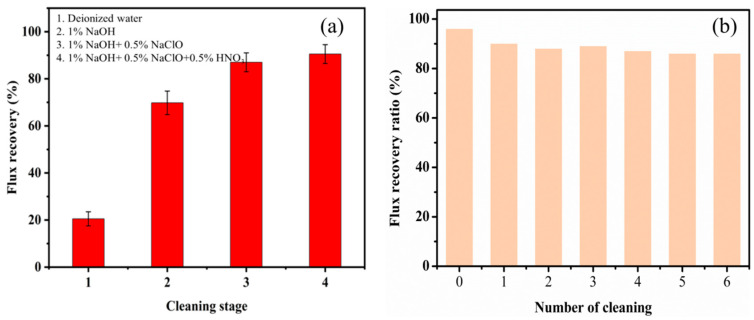
(**a**) Effects of cleaning reagents on the flux recovery ratio under different operating conditions. (**b**) Flux recovery after repeated cleanings using the fourth cleaning scheme.

As shown in Figure 8a, the contaminated membrane was cleaned with only 1% NaOH solution for 120 min, and the flux was restored to about 70%. When NaClO and NaOH solution were mixed for cleaning for 120 min and the membrane was returned to neutral with clear water, the flux was restored to about 80%. On this basis, the membrane was further cleaned with 0.5% HNO_3_ for 15 min, and the flux of pure water was restored to about 90% after washing.

Sodium hydroxide can be dispersed on the membrane surface by loosening and emulsifying, and sodium hypochlorite can be washed out using high-speed water through the gel layer on the surface of the oxidized membrane [18,34]. Therefore, the mixed solution of NaOH and NaClO improved the cleaning efficiency of the membrane used in this study. However, pollutants, such as proteins, colloids, starch, and suspended impurities, covered the metal precipitates’ surface. It is necessary to first remove the organics on the surface of the contaminated layer so that the metal ions can be exposed. In the EDX analysis of the contaminated membrane layer (Figure 6), a large amount of Ca^2+^ was found on the surface of the contaminated membrane. This may be due to the use of the lime method to clarify raw sugar, and after acid hydrolysis, there is more free Ca^2+^ in the syrup. The ZrO_2_ membrane easily networks Ca^2+^, which is difficult to remove with caustic soda and sodium hypochlorite. After shutdown, Ca^2+^ and carbon dioxide come into contact to form calcium carbonate (Table 7). Here, 0.5% HNO_3_ can be used to clean the membrane tube in 10–15 min to remove the inorganic salt precipitation. This correspondingly improves the flux. Nitric acid significantly affects the removal of metal ions, such as calcium, magnesium, and iron. This is because the precipitates are mainly attached to the membrane surface and the membrane pore’s inner surface. The dirt on the surface of the metal precipitates can be removed by cleaning with a mixture of NaClO solution and NaOH solution. Then, nitric acid and metal precipitates can be in complete contact and react, resulting in a better cleaning effect. If the acid is cleaned first, the acid solution cannot contact the metal deposit directly, and the cleaning effect may not be desirable. The flux recovery rate of the fourth cleaning scheme after 6 repeated cleanings remains at approximately 90%, as depicted in Figure 8b, indicating the suitability of this cleaning approach for purifying syrup filtered using a ceramic membrane [18,19].

Through the integration of SEM, EDX, and FTIR analyses as well as an examination of cleaning protocols for new, fouled, and cleaned membranes, it can be inferred that starches, proteins, lipids, and suspended colloidal particles in syrup along with inorganic salts, such as Ca and Fe, are the primary contributors to membrane fouling. Furthermore, this study confirms that cake layer fouling is the dominant mechanism behind membrane fouling.

## 4. Conclusions

The fouling and cleaning of a ceramic membrane with pore size of 0.05 μm were investigated during the ultrafiltration of glucose–fructose syrup. The following results were obtained from this study.

(1) At a temperature of 90 °C, a TMP of 2.5 bar, and a cross-flow velocity of 5 m/s under the total reflux operation, the flux decreased rapidly at the commencement of the experiment, but then tended to stabilize. The steady-state flux was 181.65 Lm^−2^ h^−1^. The flux of the ceramic membrane is stable and suitable for industrial applications.
(2) Membrane fouling was studied using mathematical model prediction, SEM, EDX, and FTIR. The results indicated that the dominant fouling during ultrafiltration of glucose–fructose syrup was caused by cake formation on the membrane surface, and the membrane pore blocking was a secondary pollution.
(3) Through the removal of suspended solids, the membrane-filtered glucose–fructose syrup was decolorized using activated carbon, and the filtration rate was effectively improved. The pretreatment of glucose–fructose syrup using ceramic membrane coupled with activated carbon results in low turbidity and colorless value.

Therefore, the pretreatment of glucose–fructose syrup using ultrafiltration coupled with activated carbon decolorization is feasible and efficient.

## Figures and Tables

**Table 1:** Material characteristics and module details of the membrane system used in this study.

Item	Description
Manufacturer	Jiangsu Jiuwu Hi-Tech, Nanjing, China
Membrane type	Tubular
Membrane material	ZrO_2_
Membrane support material	α-Alumina oxide
Pore size	0.05 μm
Pure water permeability	610 L/(m^2^ h bar)
Length	1016 mm
Outside diameter	30 mm
Number of channels	37

**Table 2:** Operating conditions for ultrafiltration membrane filtration of high fructose syrup.

Operating Condition	Temperature (°C)	CFV (m/s)	TMP (bar)
C1	70	5	2.5
C2	80	5	2.5
C3	90	5	2.5
C4	90	3	2.5
C5	90	4	2.5
C6	90	6	2.5
C7	90	5	1.8
C8	90	5	3.0
C9	90	5	4.0

**Table 3:** Comparison of physicochemical indices of glucose–fructose syrup feed and permeate solution (TMP = 2.5 bar; CFV = 5.0 m/s; and T = 70 °C, 80 °C, and 90 °C).

Parameters	Feed	Permeate		
		70 °C	80 °C	90 °C
Brix (%)	43.1	42.8	42.6	42.8
Turbidity (NTU)	92.15	0.68	0.70	0.66
Color (RBU)	810.8	712.1	718.6	729.4
Conductivity (μS/cm)	806	725	716	722
Light transmittance (%)	63.7	96.9	95.0	95.0
Total colloid removal rate (%)	0	69.13%	68.78%	68.43%

**Table 4:** Comparison of physicochemical indices of glucose–fructose syrup feed and permeate solution (TMP = 2.5 bar; T = 90 °C; and CFV = 3.0, 4.0, 5.0, and 6.0 m/s).

Parameters	Feed	Permeate			
		3.0 m/s	4.0 m/s	5.0 m/s	6.0 m/s
Brix (%)	44.0	43.1	43.0	42.8	43.0
Turbidity (NTU)	92.10	0.68	0.70	0.66	0.72
Color (RBU)	815.6	721.5	728.6	729.4	729.8
Conductivity (μS/cm)	801	734	741	730	726
Light transmittance (%)	62.8	95.3	95.5	95.0	95.4
Total colloid removal rate (%)	0	68.93%	68.36%	68.78%	67.90%

**Table 5:** Comparison of physicochemical indices of glucose–fructose syrup feed and permeate solution (CFV = 5.0 m/s; T = 90 °C; and TMP = 1.8 bar, 2.5 bar, 3.0 bar, and 4.0 bar).

Parameters	Feed	Permeate			
		1.8 bar	2.5 bar	3.0 bar	4.0 bar
Brix (%)	43.8	42.9	42.8	42.9	42.7
Turbidity (NTU)	92.35	0.63	0.66	0.65	0.68
Color (RBU)	818.9	722.7	729.4	726.9	728.3
Conductivity (μS/cm)	807	729	730	732	725
Light transmittance (%)	61.9	95.6	95.0	95.1	95.3
Total colloid removal rate (%)	0	68.91%	68.78%	69.28%	69.75%

**Table 6:** Summary of the fouling models.

Model	Equation
Complete pore blocking.	$\ln\left[J\right]=\ln\left[J_{0}\right]-K_{1}t$
Pore narrowing.	$\frac{1}{J_{\;}^{0.5}}=\frac{1}{J_{0}^{0.5}}+K_{2}t$
Cake filtration.	$\frac{t}{V_{f}}=\frac{1}{J_{0}A_{0}}+K_{3}V_{f}$

Note: J_0_ is the initial permeate flux (Lm^−2^ h^−1^), t is the filtration time (min), V_f_ is the volume of filtered syrup (L), A_0_ is the total membrane surface area (m^2^), J is the permeate flux of time t (Lm^−2^ h^−1^), K_1_ is the complete pore blocking constant, K_2_ is the pore narrowing constant, and K_3_ is the cake filtration constant.

**Table 7:** EDX data of the fouled and cleaned membranes.

Weight (%)	C	O	Al	Zr	Ca	Fe
Fouling layer	21.42	26.21	3.26	5.48	35.72	7.92
Fouled membrane layer	30.68	18.47	0.44	47.7	2.72	0
Fouled membrane transition layer	15.11	35.77	46.65	2.47	0	0
Fouled membrane support layer	18.48	36.47	43.98	1.07	0	0
Cleaned membrane layer	13.83	16.08	0	70.09	0	0
Cleaned membrane transition layer	14.18	31.29	44.57	5.28	0	0
Cleaned membrane support layer	9.21	37.07	51.3	2.42	0	0

## Data Availability

The raw data supporting the conclusions of this article will be made available by the authors on request.
